# Case Report: Atypical (CD34-) nevus lipomatosus cutaneous superficialis with fibroblastic nodular hyperplasia on the knee

**DOI:** 10.3389/fphys.2026.1805076

**Published:** 2026-06-03

**Authors:** Nan Huang, Yu Li, Li Li, Tingting Wang

**Affiliations:** 1 Department of Dermatology, West China Hospital, Sichuan University, Chengdu, China; 2 Laboratory of Dermatology, Clinical Institute of Inflammation and Immunology, Frontiers Science Center for Disease-related Molecular Network, West China Hospital, Sichuan University, Chengdu, China

**Keywords:** case report, concomitant diseases, diagnosis, fibroblastic nodular hyperplasia, nevus lipomatosus cutaneous superficialis

## Abstract

Nevus lipomatosus cutaneous superficialis (NLCS) is a unique hamartoma, characterized by ectopic adipose tissue in the dermis, which mainly localizes in buttock and thigh. This study elucidates a case of NLCS with fibroblastic nodular hyperplasia and reviews related literature. Herein, a 15-year-old boy was presented with subcutaneous mass consisting of multiple papules on the surface below his left knee joint for 5 years. The histopathological findings revealed ectopic mature adipose tissue and several storiform-arranged masses containing short fusiform cells and mast cells within the dermis and immunohistochemical staining indicated that CD10 was positive in the above fusiform cells. The diagnosis of nevus lipomatosus cutaneous superficialis with fibroblastic nodular hyperplasia was made. This study aims to increase awareness of this rare disease and its concomitant diseases for early recognition and proper treatment strategy.

## Introduction

Nevus lipomatosus cutaneous superficialis (NLCS) is a rare benign hamartoma of unknown etiology, characterized by papules and nodules usually in the pelvic region, with ectopic mature adipose tissue within the superficial dermis (Kwak et al., 2018; Hassab-El-Naby and Rageh, 2022). Several recent reports have revealed that NLCS may be accompanied by other diseases, including angiokeratoma, lipomatous scalp, follicular sebaceous hamartoma and so on (Sathyanarayana, 1978; Takashima et al., 2003; Anzai et al., 2015). Here, we report the first case of a 15-year-old male presented with NLCS localizing below the left knee accompanied by fibroblastic nodular hyperplasia. A systemic review of all the available literatures was performed to reveal the concomitant diseases of NLCS as well.

## Case report

A 15-year-old boy was presented with a 5-year history of subcutaneous mass consisting of multiple papules below his left knee joint. Five years ago, the patient developed subcutaneous egg-size nodules below his left knee without pain or tenderness. He went to a local hospital for help, and based on the examination of ultrasound, the diagnosis of lipoma was considered with no special treatment. Two years ago, the skin lesions gradually enlarged, and multiple papules appeared on the above skin lesions. No family history of a similar condition was reported. The patient underwent partial surgical excision. However, the skin lesions gradually enlarged.

**Figure 1:**
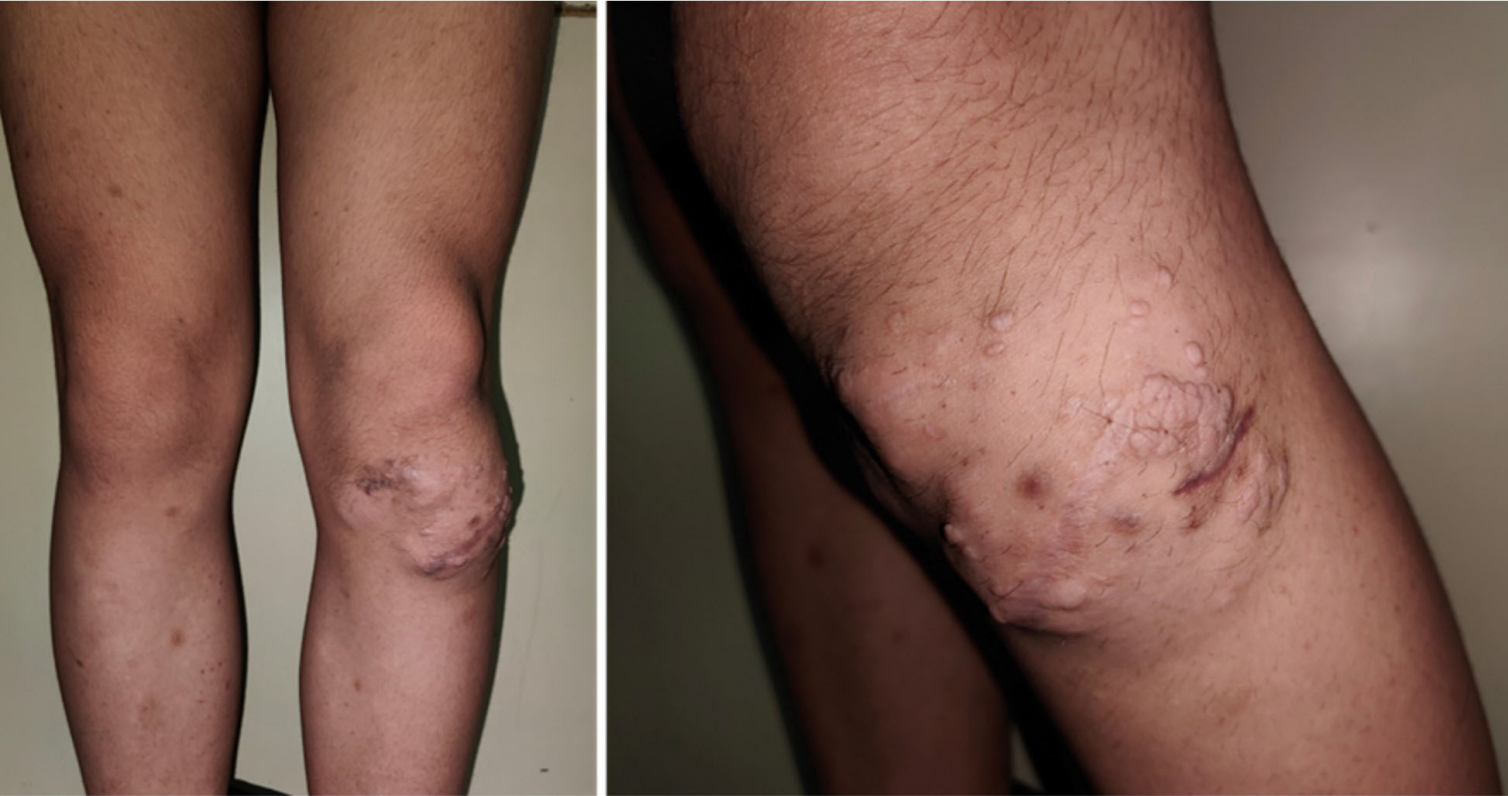
Multiple skin-colored, soft, indolent nodules, with multiple flat papules on the surface that coalesced into a plaque with cerebriform surface, below the left knee joint.

**Figure 2:**
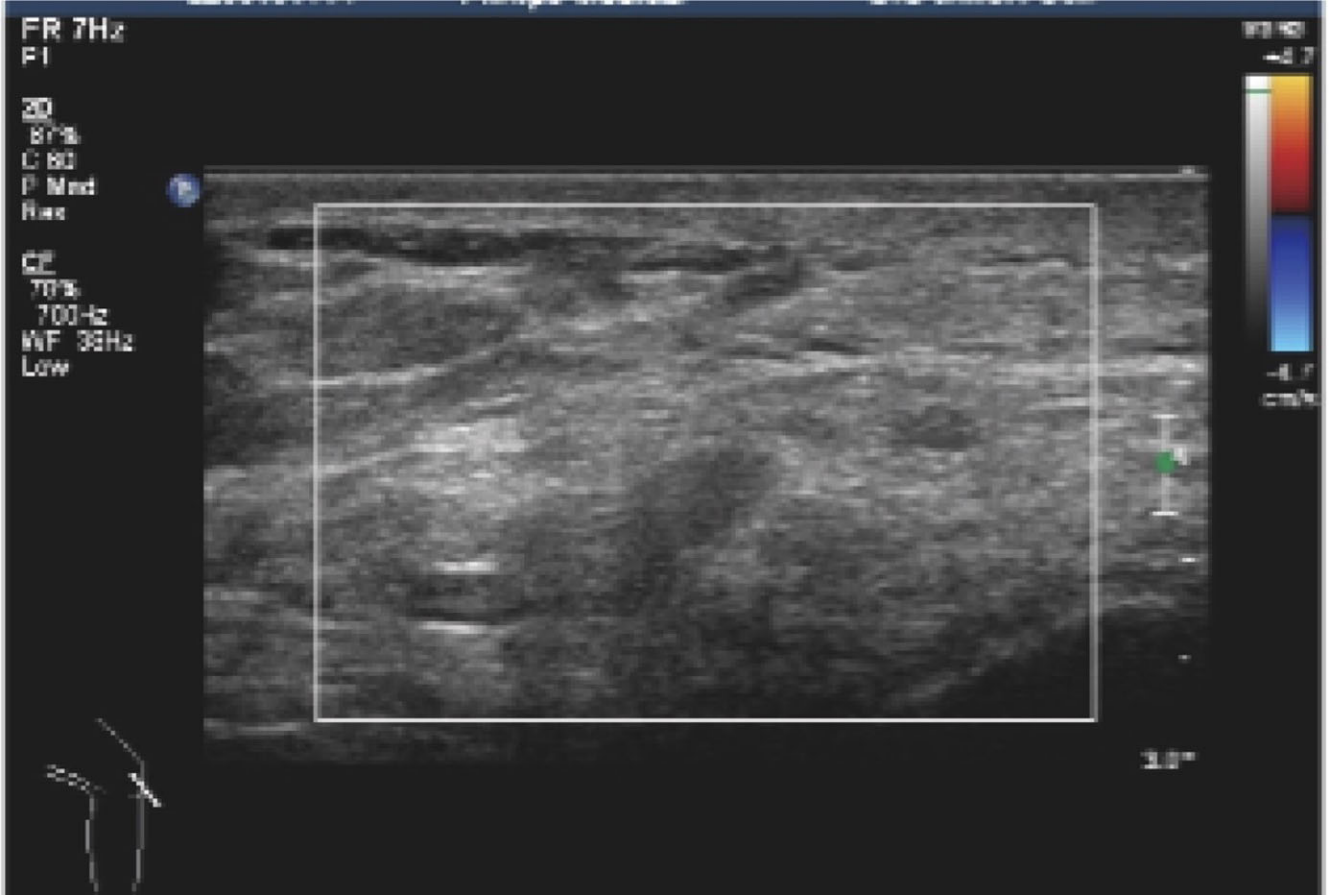
The ultrasound revealed a thickened layer of the skin and a subcutaneous layer of the left knee, with uneven and slightly strong echo and unclear boundary. The nodular hypoecho could be seen inside, and the internal blood flow signal was not rich.

**Figure 3:**
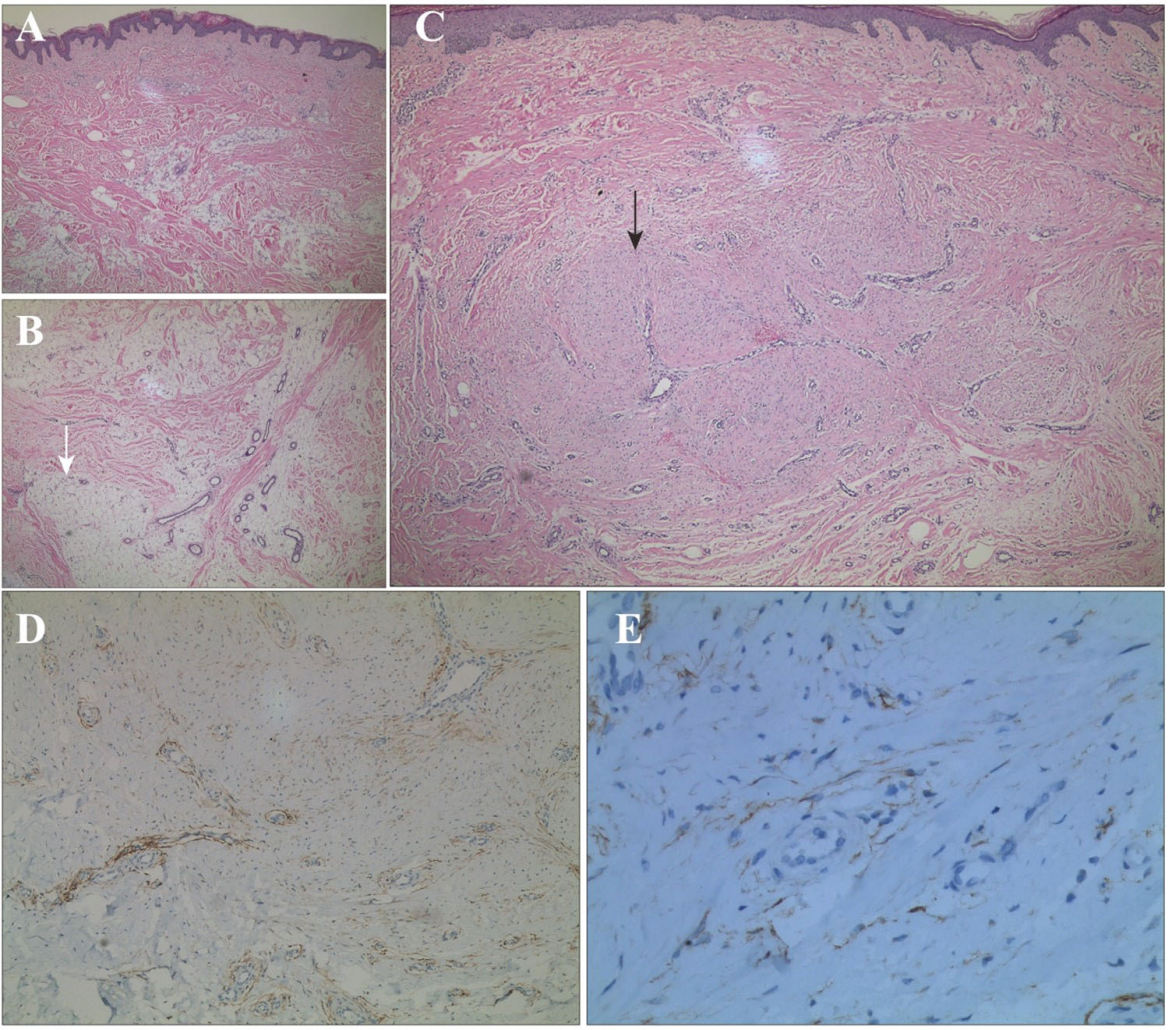
**(a)** The epidermis was mildly hyperplastic and **(b)** there was a large amount of ectopic mature adipose tissue within the dermis (white arrow). **(c)** Besides, there were many storiform arranged masses in the upper and mid-dermis (black arrow, hematoxylin and eosin, **(a-c)**: ×40 original magnification). Immunohistochemical staining showed CD10 was positive in the fusiform cells [**(d)**:×100; **(e)**:×200 original magnification].

Physical examination revealed multiple skin-colored, soft, indolent masses, with multiple flat papules on the surface that coalesced into a plaque with cerebriform surface, below the left knee joint (**Figure 1**). No ulceration or café-au-lait was observed. Besides, the ultrasound of the left knee revealed a thickened layer of the skin and a subcutaneous layer, with uneven and slightly strong echo and unclear boundary. The nodular hypoecho could be seen inside, and the internal blood flow signal was not rich (**Figure 2**). Moreover, the histopathological examination of a papule lesion revealed that the epidermis was mildly hyperplastic, and there was a large amount of ectopic maturation adipose tissue within the dermis. Also, there were several storiform-arranged masses in the dermis, which consisted of short fusiform cells and some mast cells (**Figures 3A–C**). Immunohistochemical staining indicated that CD10 was positive in the above fusiform cells, while S100, NF, EMA, Desmin, CD31, and CD34 were negative (**Figures 3D, E**). Based on the findings above, a diagnosis of nevus lipomatosus cutaneous superficialis (NLCS) with fibroblastic nodular hyperplasia was finally considered. The patient is under follow-up observation without special treatment and no changes in skin lesions were observed.

## Discussion

NLCS, first described in 1921 by Hoffman and Zurhelle, is a rare congenital or acquired benign hamartoma with an indolent, asymptomatic course (Kwak et al., 2018). The pathogenesis of NLCS remains unknown. The postulated origin of ectopic adipocytes is from adipose metaplasia during degenerative changes in connective tissue, developmental displacement of adipose tissue or perivascular differentiating lipoblasts (Kwak et al., 2018; Hassab-El-Naby and Rageh, 2022). NLCS can be presented in two variants: the multiple or classical form and the solitary form. The classical form usually appears at birth or before the age of 30 years, consisting of multiple, soft yellow to skin-colored papules or nodules located primarily in the pelvic region and thigh. The solitary form, which can appear at any skin region, is always expressed before the age of 30 years and as a skin-colored single papule or nodule. Recently, some studies elucidated that the appearance of NLCS may by unique and mimic other diseases including inverted nipple-like nodules, acrochordon, plane xanthoma and so on (Hassab-El-Naby and Rageh, 2022). The typical histopathology of NLCS is mature adipocytes embedded between the collagen bundles in the superficial dermis. Besides, proliferations, hyperkeratosis of the epidermis, proliferation of blood vessels, changes in the collagen and elastic fibers, and abnormality of follicular sebaceous glands can be present in some cases as well (Sathyanarayana, 1978; Kwak et al., 2018; Hassab-El-Naby and Rageh, 2022).

In our case, the knee joint is one of the rarest sites of NLCS, and only one case has been reported in the previous literature (Sathyanarayana, 1978). Previously reported rare locations include the vulva, perianal region, nose, and auricle (Takashima et al., 2003; Kang et al., 2007; Mansur et al., 2007; Anzai et al., 2015). In the present case, the fibroblastic nodular component consisted of short spindle cells arranged in a storiform pattern, with scattered mast cells. Immunohistochemically, these spindle cells were positive for CD10 and negative for S100, NF, EMA, desmin, CD31, and CD34. NLCS is histologically characterized by ectopic mature adipose tissue within the dermis. CD10 expression has been reported in periadnexal mesenchymal cells of normal skin and in several cutaneous mesenchymal or spindle-cell lesions, including dermatofibroma, dermatofibrosarcoma protuberans, and neurofibroma. Therefore, CD10 immunoreactivity should be interpreted in combination with the histopathological features and the broader immunohistochemical profile.

So far, there were two previous cases reporting NLCS with perifollicular fibromas. Perifollicular fibroma (PF) is a rare benign cutaneous hamartoma, characterized by proliferation of mesodermal compartment of the hair follicles. One of the histological findings of PF is increased collagen fibers arising from fibroblastic cells and similarly, fibroblastic nodular hyperplasia may be formulated through the inappropriate proliferation process of fibroblastic cells, which indicated the complex role of pluripotent embryonic fibroblasts in hamartomas (Takashima et al., 2003; Anzai et al., 2015). Therefore, we speculate the differentiation of connective tissues plays a crucial role in pathogenesis of NLCS with fibroblastic nodular hyperplasia while the underlying mechanisms still need further investigation. Other accompanied diseases reported before are summarized in **Table 1**. Even though there is little understanding of the colocalization of NLCS and other skin lesions, it may indicate a complex pathogenesis of NLCS and the importance of histopathologic examination during diagnosis.

**Table 1:** Reported cases of accompanied diseases with nevus lipomatosus cutaneous superficialis.

Case	Age	Sex	Skin sites	Concomitant disease	Therapy	Reference
1	46 years	F	Occipital area	Lipedematous scalp	None	(Mansur et al., 2007)
2	47 years	F	Sacral area	Multiple folliculosebaceous cystic hamartomas	Surgery	(Sathyanarayana, 1978)
				Dermoid cysts		
3	36 years	M	Sacral area	Folliculosebaceous cystic hamartoma	Surgery	(Kang et al., 2007)
4	33 years	M	lumbar area	Folliculosebaceous cystic hamartoma	Surgery	(Bancalari et al., 2011)
5	51 years	M	Forearm	Sebaceous trichofolliculoma	Surgery	(Bancalari et al., 2011)
6	35 years	F	Buttock	Angiokeratoma	None	(Gao et al., 2007)
7	6 years	F	Chest	Perifollicular fibromas	None	(Anzai et al., 2015)
8	10 months	F	Abdomen	Perifollicular fibrosis	/	(Takashima et al., 2003)
9	52 years	F	Back	Deep penetrating nevus	None	(Lee and Kim, 2015)
10	54 years	F	Leg	Trichofolliculoma	None	(Sakanoue et al., 2013)
11	20 years	M	Buttock and waist	Dilated hair follicles	None	(Lee et al., 2012)
12	21 years	M	Lower back	Dilated hair follicles	None	(Lee et al., 2012)
13	8 years	M	Ankle	Hypertrichosis	/	(Alsalman et al., 2020)
14	NA	M	Back	Mucinous nevus	Surgery	(Durbin et al., 2023)
15	21 years	M	Buttock and left lumbar area	Nevus sebaceous of Jadassohn	Surgery	(Turan et al., 2014)
16	6 years	M	Buttock	Calcinosis cutis and the pagetoid lipocyte spread at the epidermal–dermal junction	None	(Chopra et al., 2015)
17	10 years	F	Chest	Follicular papules and hypertrophic pilo-sebaceous units	None	(Inoue et al., 2002)
18	38 years	M	Buttock	Intramuscular lipomatosis	Surgery	(Taş and Top, 2014)
19	50 years	M	Back	Cavernous hemangioma	/	(Hann et al., 1988)
20	43 years	M	Buttock	Angiokeratoma of Fordyce	Cryotherapy	(Al-Mutairi et al., 2006)
21	31 years	F	Scalp	Pedunculated basal cell carcinoma	/	(Maeda et al., 2003)
22	49 years	F	Knee	Connective tissue naevi	/	(Orteu et al., 1996)
23	56 years	F	Scalp	Cylindroma	/	(Yu and Alowami, 2014)
24	12 years	M	Leg	Depigmented macules mimicking idiopathic guttate hypomelanosis	No	(Sendhil et al., 2013)
25	5 years	M	Buttock	Retractile testis	No	(Sendhil et al., 2013)
26	14–64 years	/	/	café-au-lait macules, scattered leukodermic spots, comedo-like pores	/	(Jones et al., 1975)

F, female; M, male; /, not mentioned.

Since NLCS usually follows a benign and asymptomatic course, treatment is generally unnecessary unless required for cosmetic concerns, symptoms, or diagnostic uncertainty. Surgical excision remains the main treatment option when intervention is indicated, and successful management with combined surgical excision and electrodesiccation has also been reported (Anzai et al., 2015). Although the pathogenesis of NLCS remains incompletely understood, this uncertainty does not substantially affect the management of typical lesions. However, further investigation is still clinically and pathologically relevant, as unusual combined lesions, such as the present case with fibroblastic nodular hyperplasia, may expand the recognized morphological spectrum of NLCS and create diagnostic overlap with other adipocytic or spindle-cell lesions. Therefore, the significance of this case lies primarily in documenting an unusual clinicopathological presentation and emphasizing the importance of careful histopathological and immunohistochemical evaluation to avoid misdiagnosis.

## Data Availability

The original contributions presented in the study are included in the article/supplementary material. Further inquiries can be directed to the corresponding author.
